# Spatially resolved osteoblast-traced transcriptomics uncovers TGF-β as a combination target with sclerostin in osteoporosis

**DOI:** 10.1038/s41413-026-00521-9

**Published:** 2026-04-02

**Authors:** Ahyoun Choi, Ji Yeon Lee, Hyejin Yoon, Xiangguo Che, Minkyeong Choi, Yongkuk Park, Kyoungseob Shin, Hyunho Lee, Jimin Park, Sung Hye Kong, Jinhyun Kim, Amos Chungwon Lee, Chan Soo Shin, Je-Yong Choi, Jungwoo Lee, Sunghoon Kwon, Sang Wan Kim

**Affiliations:** 1 Interdisciplinary Program in Bioengineering, Seoul National University, Seoul, Republic of Korea; 2 Department of Internal Medicine, Seoul National University College of Medicine, Seoul, Republic of Korea; 3 Department of Biomedical Engineering, Institute for Applied Life Sciences, University of Massachusetts, Amherst, MA USA; 4 Department of Biochemistry and Cell Biology, Cell and Matrix Research Institute, Institute for Translational Research in Dentistry, School of Medicine, Kyungpook National University, Daegu, Republic of Korea; 5 Department of Chemical Engineering, University of Massachusetts, Amherst, MA USA; 6 Department of Electrical and Computer Engineering, Seoul National University, Seoul, Republic of Korea; 7 Bio-MAX Institute, Seoul National University, Seoul, Republic of Korea; 8 Division of Endocrinology and Metabolism, Seoul National University Bundang Hospital, Seongnam, Republic of Korea; 9 Division of Data Science, College of Information and Communication Technology, The University of Suwon, Hwaseong, Republic of Korea; 10 Meteor Biotech, Co. Ltd, Seoul, Republic of Korea; 11 Division of Endocrinology and Metabolism, Seoul National University Hospital, Seoul, Republic of Korea; 12 Molecular & Cellular Biology Graduate Program, University of Massachusetts, Amherst, MA USA; 13 Division of Endocrinology and Metabolism, Boramae Medical Center, Seoul, Republic of Korea; 14 Present Address: Department of Medicine, Division of Engineering in Medicine, Brigham and Women’s Hospital, Harvard Medical School, Boston, MA USA; 15 Present Address: Botnar Institute of Immune Engineering, Basel, Switzerland

**Keywords:** Osteoporosis, Bone

## Abstract

Dynamic transitions of mature osteoblasts between active and quiescent states are essential for bone homeostasis and present a promising target for osteoanabolic therapy. However, these transitions remain poorly understood due to cellular heterogeneity and limited spatial context. Here, we employed spatially resolved osteoblast-traced transcriptomics, integrating an osteoblast-specific lineage tracing study and spatially resolved laser-activated cell sorting (SLACS), to profile osteoblast states on quiescent bone surfaces. This approach identified transforming growth factor-beta (TGF-β) signaling as a regulator of osteoblast activation. We further validated this role using single-cell RNA sequencing, in vitro functional assays, and in vivo. In a hindlimb unloading mouse model, dual inhibition of TGF-β and sclerostin enhanced bone mass and mitigated bone loss more effectively than sclerostin inhibition alone. These findings reveal a mechanistic role for TGF-β in regulating osteoblast dynamics and propose a dual-target therapeutic strategy that enhances the efficacy of anti-sclerostin treatment in osteoporosis.

## Introduction

Osteoporosis is a skeletal disorder characterized by low bone mass and microarchitectural deterioration, predominantly resulting from an imbalance between bone formation and resorption.^1^ This disruption significantly increases fracture risk, particularly in aging populations, making osteoporosis a major global public health concern with substantial socioeconomic burdens, morbidity, and mortality.^2^ Current pharmacological strategies address both aspects of bone homeostasis; anti-resorptive agents reduce osteoclastic bone resorption, whereas anabolic agents enhance osteoblast-driven bone formation.^3^ However, long-term use of these agents is constrained by adverse effects,^3,4^ highlighting the need for novel treatments that can promote rapid and effective bone regeneration.

Expanding the osteoblast population has emerged as a key strategy for enhancing bone formation and restoring the skeletal architecture. Indeed, anabolic agents that promote osteoblast proliferation and activity have demonstrated superior efficacy in reducing fracture risk and improving bone mass compared with anti-resorptive therapies.^5,6^ Notably, romosozumab, one of the most effective anabolic drugs available, promotes osteoblast expansion by inhibiting sclerostin, a key negative regulator of osteoblast functions, facilitating bone formation even in the quiescent bone metabolic state.^4,6,7^ One critical source of active osteoblasts stimulated by romosozumab is bone lining cells (BLCs), a population of quiescent osteoblasts that reside on inactive bone surfaces.^7,8^ Although BLCs can be reactivated into bone-forming osteoblasts by sclerostin inhibition, the molecular mechanisms underlying the dynamic transition between BLCs and active osteoblasts are poorly understood. This gap limits the discovery of novel therapeutic targets and hinders the design of effective combinatorial strategies.

Investigating dynamic osteoblast transitions presents several challenges. First, BLCs, which are transiently differentiated osteoblasts, lack specific histological or genetic markers, making their identification particularly difficult.^9^ Moreover, distinguishing reactivated BLCs from newly recruited osteoblasts following treatment with anti-sclerostin antibody (Scl-Ab) is challenging.^10^ Second, BLCs form a thin, spatially restricted layer along the bone surface, necessitating high-resolution techniques that preserve the spatial context while capturing their molecular signatures.

To overcome these challenges, we used spatially resolved osteoblast-traced transcriptomics, an integrative approach that combines osteoblast-specific lineage tracing with spatially resolved laser-activated cell sorting (SLACS),^11–13^, to elucidate the cellular dynamics of osteoblasts on quiescent bone surfaces. Lineage tracing allowed us to track osteoblast-lineage cells, including BLCs, and directly assess their transition into active osteoblasts by labeling specific osteoblasts and their progeny. SLACS facilitated the precise enrichment of labeled cells with minimal cellular damage to preserve their molecular integrity. Through this integrative approach, we specifically enriched and profiled labeled osteoblasts residing along the bone surfaces, identifying transforming growth factor-beta (TGF-β) signaling as a key regulator of osteoblast state transition. Further validation using single-cell RNA sequencing (scRNA-seq), along with in vitro and in vivo functional assays, confirmed the role of TGF-β in dynamic transitions between mature osteoblasts and BLCs. Finally, we demonstrated the additive therapeutic potential of TGF-β inhibition in combination with Scl-Ab treatment, providing molecular insights that could advance osteoporosis therapy.

## Results

### Spatially resolved osteoblast-traced transcriptomics captures distinct activation states of mature osteoblasts

**Fig. 1:**
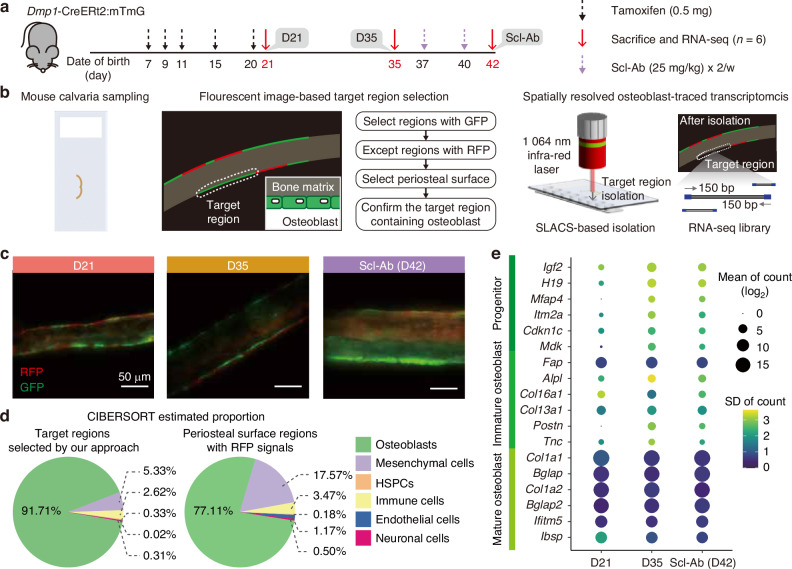
Integration of an osteoblast-lineage mouse model and SLACS enables targeted enrichment of mature osteoblasts. **a** Schematic of the *Dmp1*-CreERt2:mTmG mouse model used to label osteoblasts at different activation stages. Mice received 0.5 mg tamoxifen on postnatal days 7, 9, 11, 15, and 20, representing black dashed arrows and were euthanized on days 21 or 35, or at 6 weeks of age following Scl-Ab treatment, representing purple dashed arrows. **b** Overview of spatially resolved osteoblast-traced transcriptomics. Osteoblasts were traced by recombination from tdTomato to GFP expression. These GFP + regions were identified via fluorescent and anatomical landmarks in calvaria sections, isolated with near-infrared pulsed laser, and processed for full-length transcriptomic profiling. **c** Representative fluorescent images showing GFP and tdTomato expression in calvaria periosteum. Scale bar: 50 μm. **d** Pie charts showing deconvoluted cell type proportions using CIBERSORTx. Left: regions selected by GFP expression and periosteal surface; right: regions selected by RFP expression. **e** Dot plot of osteoblast maturity marker genes expression across groups. Dot size indicates mean expression; color intensity reflects standard deviation (SD)

To investigate osteoblast dynamics, we utilized the *Dmp1*-CreERt2:mTmG mouse model, an inducible lineage tracing system. Particularly, the *Dmp1* promoter, which is predominantly active in osteocytes and late-stage osteoblasts on bone surfaces, was chosen to monitor the transition of mature osteoblasts into BLCs-likely phenotype over a two-week interval. Upon tamoxifen injection, *Dmp1*-expressing cells switch from tdTomato to green fluorescent protein (GFP) expression through recombination, enabling the tracking of the subsequent fate transition of mature osteoblasts. Based on these dynamics and the prior studies, we designed three experimental groups with distinct states; the active state (D21), two days after the last tamoxifen injection; the inactive state (D35), two weeks after tamoxifen injection, when GFP-labeled (GFP + ) osteoblasts acquired a BLC-like phenotype; and the reactivated state (Scl-Ab), following Scl-Ab treatment, which reactivated these quiescent osteoblasts into bone-forming cells (Fig. 1a). Collectively, this lineage-tracing strategy delineates the transition of mature osteoblasts, including their reversible progression across active, inactive, and reactivated states.

To molecularly profile these states, we employed spatially resolved transcriptomics using SLACS,^11^ targeting GFP+ regions along the periosteal surface of the mouse calvaria (Fig. 1b). SLACS ablated pulsed near-infrared (NIR) laser to region-of-interest, allowing the precise enrichment of transcriptomes from spatially confined anatomical regions (Fig. S1a). To facilitate a focused analysis of GFP+ cells, we selected mouse calvaria tissue, which provides a high bone formation environment and a thin, uniform anatomical structure, enabling efficient and accurate isolation. Using this approach, we isolated the target regions selected via fluorescence imaging and confirmed morphological differences corresponding to the activity states (Fig. S1b). Specifically, GFP+ cells in the D35 group appeared thinner than those in the D21 and Scl-Ab groups, consistent with the established correlation between osteoblast activity and cell thickness^14^ (Fig. 1c). We generated transcriptome data from 39 ROIs derived from 18 male mice (6 mice per group).

To validate whether our approach specifically enriched mature osteoblasts, we performed cell-type deconvolution using CIBERSORT with publicly available scRNA-seq data^15^ and compared the results with those obtained from RFP+ regions, which were not recombined under the *Dmp1* promoter. The GFP+ isolated regions showed a highly enriched osteoblast population, with over 91% of the cells identified as osteoblasts, whereas the RFP+ regions contained approximately 77% osteoblasts (Fig. 1d and Fig. S2a). Further examination of osteoblast lineage marker gene expression revealed that mature osteoblast-associated genes, such as *Col1a1*, *Bglap*, *Ifitm5*, and *Ibsp*,^16^ were upregulated in the GFP+ regions (Fig. 1e). In contrast, RFP+ regions exhibited lower expression of *Col1a1* and *Bglap* genes (Fig. S2b). This differential expression pattern supports that GFP+ targeting selectively enriches cells at a mature osteoblasts state, whereas RFP+ regions retain a more heterogeneous and less differentiated population. Collectively, these results demonstrate that our osteoblast-targeted transcriptomic approach robustly isolates and profiles osteoblasts across functionally distinct states, that is, activation, inactivation, and reactivation, thus providing a powerful platform for dissecting osteoblast fate dynamics in vivo.

### Transcriptomic profiles define osteoblast states

**Fig. 2:**
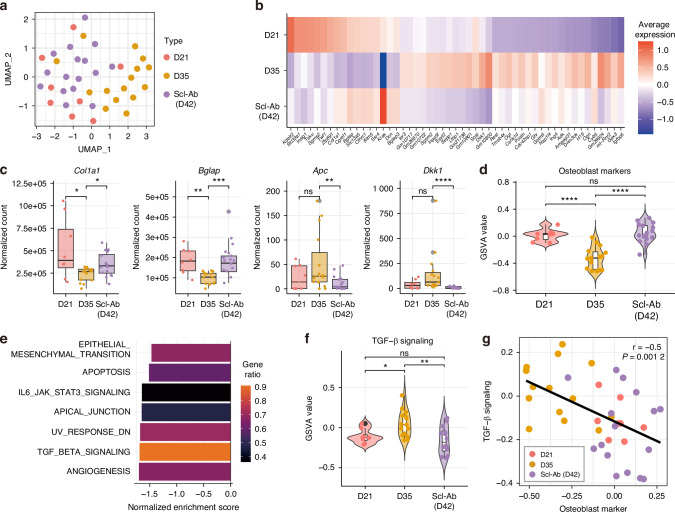
Transcriptomic profiles reveal that TGF-β signaling is negatively correlated with osteoblast activity. **a** Uniform manifold approximation and projection (UMAP) plot of all isolated regions, colored by group. **b** Heatmap showing the average expression of the top 20 up- and downregulated genes compared to D35. **c** Boxplots of osteogenesis and Wnt signaling inhibitor gene expression. Box = IQR; line = median; whisker = 1.5 × IQR. The Wilcoxon rank-sum test is used for statistics. **d** Violin plots of osteoblast marker gene set scores based on gene set variation analysis (GSVA). Each point = single region; Wilcoxon rank-sum test was used for statistics. **e** Bar plots of normalized enrichment scores of significant pathways identified by gene set enrichment analysis (GSEA). Color represents gene ratio with each pathway, representing the proportion of genes detected in the dataset relative to the total number of genes in each canonical gene set. **f** Violin plots of TGF-β signaling activity scores derived from GSVA. Each data point represents an individual region. The Wilcoxon rank-sum test was used for statistics. **g** Scatter plot showing a negative correlation between TGF-β signaling activity and osteoblast marker scores. Pearson correlation coefficient (*r*) and *P*-value are calculated across regions. *P < 0.05; **P < 0.01; ***P < 0.001; ****P < 0.000 1; ns not significant

Next, we characterized the three groups, D21 (active), D35 (inactive), and Scl-Ab (reactivated), using Uniform manifold approximation and projection (UMAP) visualization and differential gene expression analysis. Despite being sacrificed a week later than the D35 group, the Scl-Ab group displayed profiles closely resembling those of the D21 group, in contrast to the distinct profiles observed in the D35 group (Fig. 2a, b). Specifically, osteogenic genes such as *Col1a1* and *Bglap* were significantly upregulated in the D21 and Scl-Ab groups compared to those in the D35 group (Fig. 2b, c). Inhibitors of Wnt signaling, including *Dkk1* and *Apc*, which negatively regulate bone formation, were significantly downregulated in the Scl-Ab group, whereas the D35 group exhibited elevated expression of inactivation-related gene such as *Sost* (Fig. 2b, c). Additionally, we evaluated scores of osteoblast differentially expressed (DE) genes obtained from the previous publications,^15,17,18^, including osteogenic (*Col1a1*, *Bglap*) and osteoblast differentiation-related (*Runx2*, *Dmp1*, *Sp7*), and mineralization-related genes (*Alpl*, *Ibsp*, *Spp1*) (Fig. S3a). With all three different osteoblast DE genes, the osteoblast score was significantly higher in the D21 and Scl-Ab groups than in the D35 group (Fig. 2d). These results indicate that our three groups effectively represent the active, inactive, and reactivated states of osteoblasts, with distinct transcriptional and functional characteristics corresponding to each state.

### TGF-β signaling is associated with osteoblast state dynamics

To elucidate the mechanisms underlying osteoblast state regulation, we performed Gene Set Enrichment Analysis (GSEA) comparing the Scl-Ab group to the D35 group. GSEA identified several significantly regulated pathways (Fig. 2e). Then, we observed Pearson correlation of each pathway expression score with the osteoblast marker scores (Fig. 2f, g and Fig. S3b). Among these, three pathways—TGF-β signaling, UV response DN, and apical junctions—showed significant negative correlations, suggesting their potential association with osteoblast state transition. Since UV response DN and apical junction represent broad or indirect cellular responses with limited regulatory targets, we focused on the downregulation of TGF-β signaling, which exhibited the lowest normalized enrichment score and the highest gene ratio among the three pathways. Furthermore, TGF-β signaling is a canonical, drug-modulable pathway known to suppress osteogenic differentiation by inhibiting Runx2 activity,^19^ and its inhibition has been reported to promote bone formation in previous studies.^20–24^ Based on these findings, we hypothesized the role of TGF-β signaling in regulating osteoblast active states.

We further examined TGF-β signaling at the protein level following modulations of sclerostin. Using an osteoblast cell line, MC3T3-E1 cells, we observed upregulation of phosphorylated Smad3 (p-Smad3), a key signal transducer of TGF-β signaling, following recombinant sclerostin treatment (Fig. S4). C57B1/6 J mice were treated with Scl-Ab for either 1 or 4 weeks (Fig. S5a), and tissue-level changes in TGF-β signaling were measured via p-Smad3 immunohistochemistry (IHC) in femoral sections. To establish reference levels of p-Smad3 expression, we used *Sost* knockout (KO) and *SOST* transgenic (SOST-TG) mice, which represent minimal and maximal sclerostin activity, respectively. As expected, SOST-TG mice exhibited the highest number of p-Smad3+ cells in bone matrix, indicating enhanced TGF-β signaling with sclerostin overexpression. Notably, Scl-Ab treatment significantly reduced the number of pSmad3+ cells in the bone matrix (Fig. S5a–c). This reduction was consistently observed across multiple age groups in tibial sections (Fig. S5d–h), demonstrating a reliable decrease in TGF-β signaling. These findings support that modulation of TGF-β signaling is involved in the osteoblast-activating response triggered by Scl-Ab.

### scRNA-seq reveals downregulation of TGF-β signaling in active osteoblasts

**Fig. 3:**
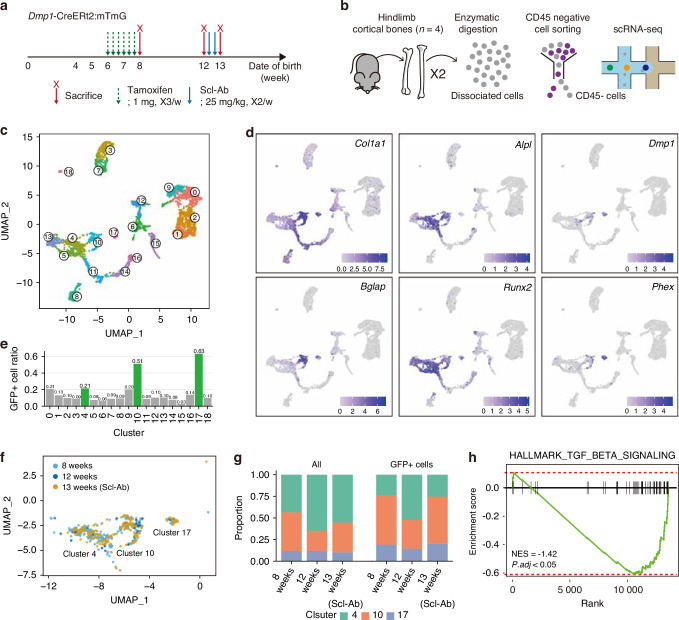
scRNA-seq shows downregulated TGF-β signaling in active osteoblasts. **a** Experimental timeline for scRNA-seq using the *Dmp1*-CreERt2:mTmG mouse model across activation states and Scl-Ab treatment. **b** Overview of scRNA-seq workflow. Hindlimb bones were dissociated, CD45- cells were sorted, and subjected to scRNA-seq (*n* = 4). **c** UMAP of 3 968 cells, with clusters identified by differential gene sets. Two osteoblast-related clusters (clusters 4 and 10) and one osteocyte cluster (cluster 17) were annotated. Color indicates the respective cluster. **d** Feature plots of osteoblast markers: *Col1a1*, *Bglap*, *Alpl*, *Runx2*, *Dmp1*, and *Phex*. **e** Bar plots of GFP+ cells proportions by cluster. Green bars indicate the highest proportions. **f** UMAP of osteoblast-lineage clusters, colored by experimental group. **g** Stacked bar plots showing cell distribution across clusters. Left: within total cells; right: within GFP+ cells. **h** GSEA enrichment plot of TGF-β signaling

Next, we performed scRNA-seq analysis to characterize the cellular landscape associated with Scl-Ab treatment. To obtain sufficient cell numbers, we isolated cells from four paired femurs and tibiae of lineage tracing mice sacrificed at 8, 12, and 13 weeks, using serial enzymatic dissociation. Non-hematopoietic cells were subsequently enriched by sorting CD45-negative populations using fluorescence-activated cell sorting (FACS) prior to scRNA-seq library preparation (Fig. 3a, b). GFP+ cells were computationally labeled based on EGFP transcript expression using a custom murine reference genome containing the EGFP sequence. After integrating the datasets from each group, we obtained 3,968 cells distributed across 19 clusters and annotated them based on canonical marker gene expression^16,25,26^ (Fig. 3c and Fig. S6). Among these, we focused on three clusters identified as osteoblast-lineage cell types: clusters 4 and 10, representing osteoblast-related states, and cluster 17, representing osteocytes. Clusters 4 and 10 expressed hallmark osteoblast markers, such as *Col1a1*, *Alpl*, and *Runx2*. Notably, Cluster 10 displayed a significantly higher expression of *Col1a1* and *Bglap*, indicating mature osteoblasts actively contributing to matrix production and mineralization. In contrast, cluster 4 cells exhibited lower expression of these genes and higher expression of *Spp1*, *Tnc*, *Mmp13*, and *Vdr*, suggesting a state characteristic of less active osteoblasts, including BLCs. Cluster 17 was distinct in its expression of *Dmp1* and *Phex*, which was consistent with that in osteocytes (Fig. 3d and Fig. S7a). GFP+ cells, which are indicative of lineage tracing, were enriched in these three clusters, further supporting their assignment to the osteoblast lineage (Fig. 3e).

Next, we assessed the effects of Scl-Ab treatment on these osteoblast-lineage clusters (Fig. 3f). Notably, Scl-Ab led to a shift in the proportions of osteoblast-related clusters, characterized by an increase in the proportion of cluster 10 and a corresponding decrease in cluster 4 compared to the 12 weeks group, particularly among GFP+ cells (Fig. 3g and Fig. S7b). This shift highlights the role of Scl-Ab in promoting osteoblast activation and enhancing the proportion of cells involved in matrix production and mineralization. To further investigate the transcriptional profiles of active osteoblasts, we performed GSEA on the GFP+ cells in these three key clusters. Regardless of Scl-Ab treatment, TGF-β signaling was significantly downregulated in active osteoblasts compared to other osteoblast-lineage cells (Fig. 3h and Fig. S7c). This finding indicates that the activation state of osteoblasts is intrinsically linked to the suppression of TGF-β signaling, highlighting a potential regulatory mechanism governing osteoblast function.

### In vitro study reveals the role of TGF-β signaling in osteoblast regulation

**Fig. 4:**
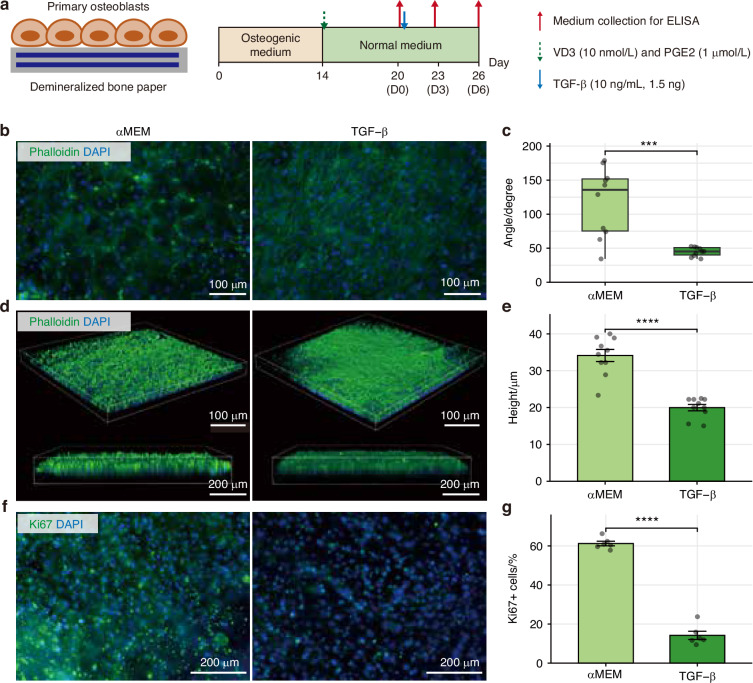
TGF-β drives the transition of active osteoblasts into BLC-like cells in DBP-based bone organoid. **a** Experimental timeline of osteoblast culture on DBP and treatment with VD3, PGE2, or TGF-β. **b** Representative immunofluorescence images, showing actin-phalloidin (green) and nucleus-DAPI (blue). Scale bar = 100 μm. **c** Box plots of actin angles (*n* = 8). Wilcoxon rank-sum test was used for statistics. **d** Representative confocal images, showing actin-phalloidin (green) and nucleus-DAPI (blue). **e** Bar plot of cell height quantified from confocal images (*n* = 8). A *t*-test was used for statistics. **f** Representative immunofluorescence images, showing Ki67 (green), and nucleus-DAPI (blue). **g** Bar plot of the Ki67-positive cell ratio of stained cells (*n* = 8). Proportions of Ki67-positive cells were calculated as the number of Ki67-positive cells divided by the number of DAPI-positive cells. Each point represents individual well. A *t*-test was used for statistics. ***P < 0.001; ****P < 0.000 1

Next, we investigated the in vitro effects of TGF-β signaling on the regulation of osteoblast status. To recapitulate the in vivo functions of osteoblasts, we cultured bone organoids on demineralized bone paper (DBP).^27–30^ Primary osteoblasts isolated from mouse femurs and tibiae were seeded onto DBP and cultured in osteogenic differentiation medium for two weeks, during which they actively deposited minerals and adopted a quiescent, BLC-like phenotype. These inactive osteoblasts were then stimulated into active states with vitamin D3 (VD3) and prostaglandin E2 (PGE2) for six days (Fig. 4a). Upon withdrawal of the stimuli, osteoblasts gradually reverted to an inactive, BLC-like phenotype, as previously reported.^28^ We analyzed the morphological changes using actin staining. TGF-β treatment resulted in osteoblasts aligning more uniformly along actin filaments, whereas the control group exhibited a broader range of alignment angles (Fig. 4b, c). Confocal 3D-reconstituted images showed that TGF-β-treated osteoblasts formed flatter cellular assemblies with significantly reduced vertical thickness, adopting a more BLC-like morphology (Fig. 4d, e). Moreover, given that cellular proliferation reflects the activity of osteoblasts,^27^ we performed immunofluorescence staining of the mitotic marker Ki67, revealing a significant reduction in Ki67-positive cells upon TGF-β treatment (Fig. 4f, g). In addition, time-course enzyme-linked immunosorbent assay (ELISA) analysis of key regulatory molecules, receptor activator of nuclear factor kappa-B ligand (RANKL) and osteoprotegerin (OPG), revealed that TGF-β treatment significantly accelerated the recovery of OPG secretion and the decline of RANKL secretion compared to the untreated controls (Fig. S8). Collectively, these results demonstrate the significance of TGF-β in promoting osteoblast transition into BLCs.

Furthermore, to complement the osteoblast-only in vitro system, we performed osteoblast-osteoclast co-culture experiments (Fig. S9). Primary osteoblasts were cultured on DBP for two weeks, followed by co-culture with bone marrow-derived monocytes (BMMs) with stimulation of VD3 and PGE2. In this co-culture system, TGF-β treatment suppressed osteoclast differentiation, indicating that TGF-β signaling influences not only osteoblast activity but also cellular processes associated with bone resorption.

### Lineage tracing mouse models reveal the role of TGF-β in cellular dynamics of osteoblast differentiation into BLC and BLC reactivation

**Fig. 5:**
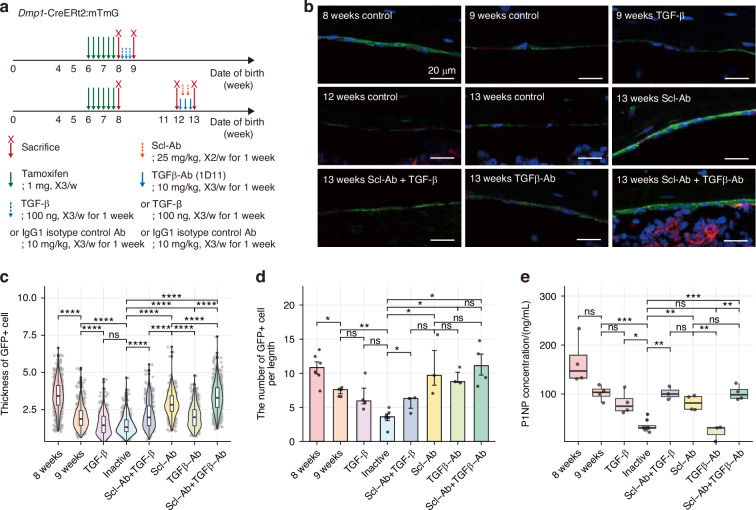
Lineage tracing reveals that TGF-β regulates osteoblast-BLC conversion and reactivation. **a** Experimental timeline of tamoxifen pulse and TGF-β/TGFβ-Ab administration in *Dmp1*-CreERt2:mTmG mice. **b** Representative confocal images of femoral periosteum showing GFP (green), tdTomato (red) signals, and DAPI (blue). Scale bar = 20 μm. **c** Violin plot of GFP+ cell thickness. Each point represents individual cells. The Wilcoxon rank-sum test was used for statistics. **d** Bar plot of GFP+ cells per unit length. Cell numbers were determined from three comparable sections per mouse, with eight fields (400× magnification) analyzed per section. The Wilcoxon rank-sum test was used for statistics. **e** Box plot of serum P1NP levels. A *t*-test was used for statistics. Group sizes: 8 weeks (*n* = 6), 9 weeks (*n* = 5), TGF-β (*n* = 4), Inactive (12 weeks control + 13 weeks control; *n* = 6), Scl-Ab+TGF-β (*n* = 3), Scl-Ab (*n* = 3), TGFβ-Ab (*n* = 4), and Scl-Ab+TGFβ-Ab (*n* = 5). *P < 0.05; **P < 0.01; ***P < 0.001; ****P < 0.000 1; ns not significant

To investigate the in vivo effects of TGF-β signaling on BLC reactivation, we used *Dmp1*-CreERt2:mTmG mice with administration of either TGF-β or TGF-β blocking antibody (TGFβ-Ab) in combination with Scl-Ab (Fig. 5a). At postnatal week 8 (two days after the last tamoxifen injection), GFP-expressing plump osteoblasts were observed on the endosteal surface of the femur, estimated to be in an active state, using the cell thickness as a proxy measure to assess osteoblast activation. Over time, these GFP+ cells flattened, and their number declined, reflecting their conversion into BLCs, as previously reported.^7,8^ Treatment with TGF-β accelerated this conversion, resulting in thinner and fewer GFP+ cells compared to the 9-week control group. Similarly, administering TGF-β alongside Scl-Ab at postnatal week 12 attenuated the effects of Scl-Ab, reducing the activation of GFP+ cells. Conversely, administration of neutralizing TGFβ-Ab, either alone or in combination with Scl-Ab, resulted in a significant increase in cuboidal GFP+ cells on the endosteal surface, while vehicle-treated mice exhibited only flat GFP+ cells. Although TGFβ-Ab alone modestly promoted the conversion of BLCs into osteoblasts compared to vehicle treatment, the combination of Scl-Ab and TGFβ-Ab significantly enhanced the thickness of GFP+ cells compared to Scl-Ab alone (Fig. 5b, c). Quantitative analysis confirmed that TGFβ-Ab, particularly in combination with Scl-Ab, promoted BLC activation and increased the thickness and number of GFP+ cells (Fig. 5c, d). However, neither TGF-β nor TGFβ-Ab induced significant changes in serum P1NP (a marker of bone formation) levels (Fig. 5e). Collectively, these results support the role of TGF-β in the context of mature osteoblast differentiation and BLC reactivation, demonstrating morphological changes associated with bone-forming activity.

### Dual inhibition of TGF-β and sclerostin under hindlimb unloading shows additive bone formation

**Fig. 6:**
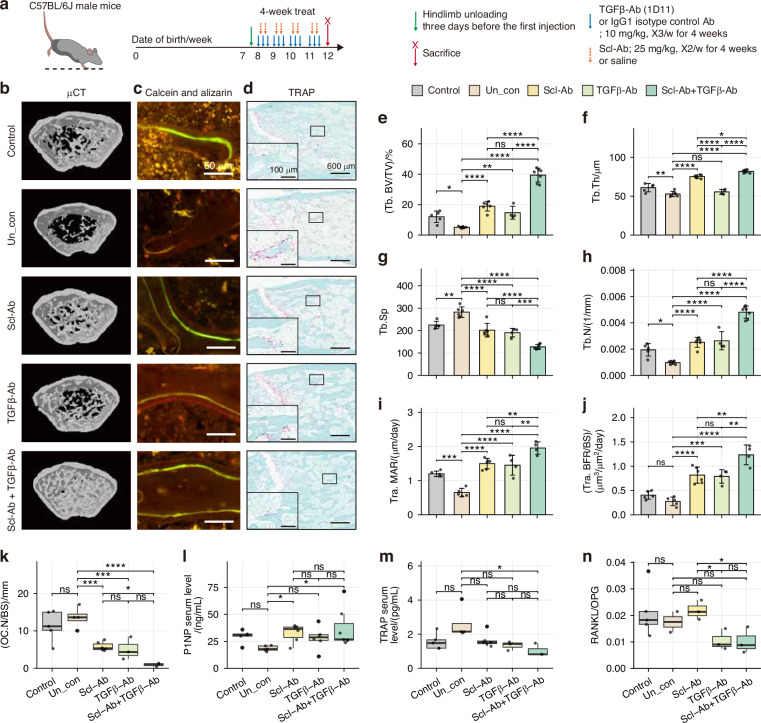
Dual inhibition of TGF-β and sclerostin increases bone mass in a hindlimb unloading model. **a** Experimental timeline of hindlimb unloading using C57B1/6 J mice and treatment with TGFβ-Ab, Scl-Ab, or both. Mice euthanized at week 12. **b** Representative μCT images of femoral diaphysis. **c** Representative confocal images of trabecular bone showing calcein (green), alizarin (red) signals, and DAPI (blue). Scale bar = 100 μm. **d** Representative images of TRAP-stained femur sections; violet = TRAP-positive, green = counterstain. Scale bar = 200 μm. **e**–**h** Bar plot of trabecular bone volume/total volume (Tra. BV/TV, **e**), trabecular thickness (Tb. Th, **f**), trabecular separation (Tb. Sp, **g**), and trabecular number (Tb. N, **h**) from μCT. Data = mean ± standard deviation. **i**, **j** Bar plot of mineral apposition rate (Tra. MAR, **i**) and trabecular bone formation rate per bone surface (Tra. BFR/BS, **j**) from labeling images. Data = mean ± standard deviation **k** Box plot of TRAP+ osteoclasts per bone surface (OC.N/BS), measured through ImageJ from TRAP-stained slides. **l**–**n** Box plot of serum P1NP (**l**) and TRAP (**m**) levels, and RANKL/OPG ratio (**n**). Group sizes: Control (*n* = 5), Un_con (*n* = 5), Scl-Ab (*n* = 6), TGFβ-Ab (*n* = 6), and Scl-Ab+TGFβ-Ab (*n* = 7). Each point represents an individual mouse. ANOVA was used for statistics; *P < 0.05; **P < 0.01; ***P < 0.001; ****P < 0.000 1; ns not significant

To further investigate the effects of TGF-β inhibition on bone formation in the setting of pathologic bone loss, we employed a mouse model of hindlimb unloading.^31^ C57B1/6 J mice underwent unloading three days prior to postnatal week 8 and were subsequently treated with TGFβ-Ab, Scl-Ab, or both for four weeks (Fig. 6a). Micro-computed tomography (μCT) analysis of trabecular bone in the femoral metaphysis revealed a substantial reduction in bone mass in unloaded mice compared to non-unloaded controls. Notably, dual administration of Scl-Ab and TGFβ-Ab resulted in a marked increase in trabecular bone volume fraction (BV/TV), thickness, and number, along with a significant decrease in trabecular separation, compared to either treatment alone. In contrast, TGFβ-Ab alone induced only a modest increase in bone mass (Fig. 6b, e–h and Fig. S10a, b). These results indicate that co-inhibition of sclerostin and TGF-β enhances bone mass more effectively than individual treatments.

Dynamic histomorphometric analysis showed a significant increase in both endocortical and trabecular bone formation following co-inhibition of sclerostin and TGF-β, surpassing the effects observed following Scl-Ab treatment alone (Fig. 6c, i, j and Fig. S10c–e). Quantification of osteoblast number using toluidine blue staining showed co-inhibition increased osteoblast counts to a level comparable to that observed with Scl-Ab treatment (Fig. S11a, b). Additionally, tartrate resistant acid phosphatase (TRAP) staining, indicative of bone resorptive activity, demonstrated that co-administration significantly reduced resorption in the trabecular bone, as evidenced by a marked decrease in osteoclast number (Fig. 6d, k). Further analysis of bone turnover markers revealed that TGF-β inhibition led to a modest increase in P1NP levels while significantly decreasing TRAcP 5b levels and the RANKL/OPG ratio (Fig. 6l–n) These findings indicate dual actions, which simultaneously stimulate bone formation and suppress resorption, consistent with the effects of Scl-Ab. Furthermore, combined administration of TGFβ-Ab and Scl-Ab not only enhanced bone formation activity but also suppressed bone resorption activity, ultimately leading to a significant increase in bone mass, as quantified in μCT analysis. This increase surpassed the effects of monotherapy. IHC analysis of femoral sections for p-Smad3 and RANKL revealed that co-administration resulted in a significant reduction in p-Smad3 expression and a corresponding increase in RANKL expression, respectively (Fig. S11c–f). Collectively, these findings suggest that TGF-β inhibition increases bone mass through mechanisms distinct from Scl-Ab, extending beyond BCL reactivation to also suppress bone resorption. This highlights the potential of combining TGF-β inhibition with Scl-Ab as a highly effective therapeutic strategy for osteoporosis treatment.

## Discussion

In this study, we investigated the cellular dynamics of mature osteoblasts and BLCs using spatially resolved osteoblast-targeted transcriptomics, combining a lineage tracing mouse model with SLACS-based microanatomic enrichment. A comprehensive functional approach revealed that TGF-β plays an inhibitory role in osteoblast activity by promoting quiescence in mature osteoblasts. Furthermore, pharmacological inhibition of TGF-β in combination with sclerostin blockade significantly enhanced trabecular bone mass in the mouse model of hindlimb unloading through an additive effect.

A comprehensive understanding of the transcriptomic profiles of BLCs has been challenging due to the lack of histological markers and their thin, localized presence. Advances in RNA-seq technologies, such as scRNA-seq^16,32^ and laser capture microdissection (LCM),^26^ have enabled the investigation of diverse osteoblast populations, revealing heterogeneity and distinct differentiation trajectories. However, these advances fall short of capturing the cellular characteristics of BLCs, largely due to the loss of spatial contexts, particularly their quiescent bone surface localization, or insufficient enrichment resolution, respectively. We addressed these limitations by employing an integrative approach. While lineage tracing mouse models allow us to track mature osteoblasts and their progeny, SLACS pushes the target regions by ablating the sacrificial layer within target regions, minimizing damage while achieving high-resolution isolation down to single cells.^33^ The fidelity and robustness of SLACS-based isolation is demonstrated in the previous study, showing preservation of genomic integrity more effectively than LCM^34^ and facilitating deeper analysis.^11–13^ By utilizing these advanced techniques, we successfully compared the transcriptomic profiles of osteoblasts across different states, overcoming the inherent challenges posed by the rarity and spatially constrained distribution of these cell populations within bone tissue.

Building on this framework, we delineated the transcriptomic features associated with the cellular dynamics of osteoblasts. First, we observed that reactivated osteoblasts exhibited transcriptomic profiles that were highly similar to those of mature osteoblasts, consistent with their previously described morphological resemblance.^7,8^ While earlier investigations of reactivated osteoblasts are largely based on morphological observations, our findings demonstrate that sclerostin inhibition restores the transcriptional profiles of these cells toward those of mature osteoblasts. Furthermore, we identified transcriptomic signatures associated with putative BLCs using scRNA-seq and lineage tracing. GFP+ cells within the inactive osteoblasts cluster, derived from *Dmp1*-expressing cells or their descendants, were considered potential BLCs. These cells showed upregulation of *Spp1*, *Tnc*, *Mmp13*, and *Vdr* genes, along with downregulation of *Col1a1* and *Bglap* expression, suggesting this pattern as a potential marker for BLCs. Although similar transcriptional profiles have been previously reported and interpreted as osteoprogenitors,^18,25^ other studies have indicated that BLC shared characteristics with mesenchymal stem/progenitor cells.^16,32^ Lineage tracing analysis further supported the identification of these cells as BLCs. However, functional validation of these gene sets is necessary, and further studies are required to elucidate the physiological roles of BLCs.

This study suggests a crosstalk between sclerostin and TGF-β. Sclerostin, secreted by osteocytes, is known to be a key regulator of osteoblast quiescence, and our findings indicate that it also might stimulate TGF-β signaling. While previous studies have reported that TGF-β signaling modulates sclerostin expression,^35,36^ we observed that sclerostin modulation, whether through treatment with Scl-Ab or genetic manipulation of *Sost* (overexpression or knockout), was positively correlated with p-Smad3 expression. More research is necessary to clarify the relationship between sclerostin and TGF-β.

In the hindlimb unloading model, TGF-β blocking antibodies exhibited a dual action by enhancing bone formation while suppressing bone resorption. It remains to be determined whether this represents a general mechanism or one that is specific to the unloading condition. In fact, increased bone mass following TGF-β inhibition has been widely reported, with multiple underlying mechanisms proposed. Studies using genetic mouse models, including conditional deletion of Tgfbr2 in mature osteoblasts, have demonstrated increased bone mass and potentiation of PTH-induced bone formation.^37^ In wild-type mice, treatment with either a TGF-β–neutralizing antibody or a type I receptor kinase inhibitor similarly enhanced bone formation while reducing bone resorption.^21,22^ Interestingly, in a spinal cord injury–induced bone loss model, TGF-β inhibition primarily stimulated bone formation,^20^ whereas in renal osteodystrophy it predominantly suppressed bone resorption.^36^ These findings collectively suggest that TGF-β inhibition can increase bone mass through context-dependent mechanisms.

In addition, in osteogenesis imperfecta (OI)—a rare connective-tissue disorder characterized by abnormalities in type I collagen—treatment with TGF-β blocking antibodies improved bone mass and mechanical strength in both preclinical and clinical studies, further supporting the therapeutic relevance of TGF-β targeting.^23,38,39^ Notably, we found that neutralizing TGF-β in combination with sclerostin blockade under conditions of bone loss resulted in a greater increase in trabecular bone mass than either monotherapy alone. This suggests that sclerostin inhibition and TGF-β inhibition act through distinct, partially complementary pathways. To date, our findings indicate that sclerostin inhibition contributes more robustly to bone formation, whereas TGF-β inhibition exerts a comparatively stronger effect on suppressing bone resorption. Our serum ELISA data and in vivo lineage-tracing analyses support this interpretation. Together, these results highlight the potential for dual-action approaches or combination therapies with romosozumab in translational settings. Further work is needed to elucidate additional pathways through which TGF-β inhibition—alone or in combination with sclerostin inhibition—may enhance bone mass.

This study had several inherent limitations. Scl-Ab was administered short-term to young mice to preserve their RNA integrity by avoiding decalcification. Although we confirmed that our experimental groups exhibited characteristic morphology and expression of osteogenic genes, physiological responses may differ after skeletal maturation. Another limitation is that our findings were based on a lineage tracing mouse model driven by the *Dmp1* promoter. To validate the generalizability of our results, further studies could incorporate models that use promoters related to other osteoblast-specific promoters. A further limitation is that we did not directly track osteoblasts with genetically downregulated TGF-β signaling in vivo, suggesting a potential focus for further investigation. Additionally, other anabolic agents, such as PTH or abaloparatide, may promote BLC conversion through distinct mechanisms, potentially revealing alternative pathways for BLC reactivation. We also did not assess the effects of TGF-β modulation in other organs. Given the widespread role of TGF-β signaling throughout the body, its inhibition could lead to unintended side effects in pharmacological applications. Nonetheless, our data demonstrates that concurrent inhibition of TGF-β and sclerostin effectively increases bone mass under conditions resembling osteoporosis, supporting its potential therapeutic relevance. Finally, TGF-β exhibited distinct functional effects on bone resorption between the in vivo and in vitro systems. Whereas TGF-β inhibition reduced bone resorption in vivo, TGF-β treatment in the in vitro co-culture system suppressed osteoclast differentiation. Although further studies are needed to elucidate the basis of this divergence, it likely reflects fundamental differences between the complex in vivo bone microenvironments and in vitro culture conditions. Despite these limitations, the in vitro system remains valuable for validating osteoblast-specific responses. Incorporating humanized bone organoids into mouse models in future studies could further enhance the translational value of this strategy, aligning with the shift toward human organoid-based preclinical research under the FDA Modernization Act 2.0.^40–42^

In conclusion, our study establishes a connection between TGF-β signaling and the interconversion of BLCs and active osteoblasts, providing the transcriptomic characterization of BLC reactivation. We demonstrate a significant additive increase in bone mass resulting from the combined inhibition of TGF-β and sclerostin. Taken together, these findings suggest that TGF-β signaling could be a promising therapeutic target for conditions involving excessive bone loss.

## Materials and methods

### Mice

To trace osteoblast lineage cells in a temporally controlled manner, *Dmp1*-CreERt2^7,8^ (provided by Dr. Hank Kronenberg, Massachusetts General Hospital) was crossed with mTmG reporter mice (The Jackson Laboratory, USA). The membrane-targeted EGFP (mG), which is expressed following CreERt-mediated gene recombination with the deletion of membrane-targeted tdTomato (mT), was used to detect the recombination induced by tamoxifen injection, because the mT cassette, located just upstream of the mG cassette, is removed by the Cre-recombinase. mG can then be expressed continuously in the targeted cells and their progeny, enabling tracking of mature osteoblasts or their descendants.

Male mice were used for spatially-resolved osteoblast-traced transcriptomics, scRNA-seq, and in vivo functional studies, each with different administration schedules.

For spatially-resolved osteoblast-traced transcriptomics, the experimental schedule was designed to remove the decalcification process to conserve RNA integrity. To induce transient nuclear translocation and CreERt2-mediated gene recombination, 0.5 mg Tamoxifen (T5648, Merck, Germany) diluted in corn oil was administered three times weekly from postnatal weeks 1 and 2. Mice were injected subcutaneously with Scl-Ab (25 mg/kg, Amgen Inc., USA; Belgium) twice a week at postnatal week 5. To determine the fate of mature osteoblasts in vivo, mice were euthanized on postnatal weeks 3, 5, and 6 (baseline, prior to, and following Scl-Ab at the indicated time). Calvarias were dissected, embedded in OCT compound, and sectioned at 8 μm thickness using a cryostat (CM1850, Leica, Germany).

For scRNA-seq, the experimental schedule was delayed by 4 weeks compared to that for SLACS-based RNA-seq, with 1 mg Tamoxifen injection and the euthanasia schedules on postnatal week 8, 12, and 13. Then, dissected bone tissues were processed through scRNA-seq preparation.

For an in vivo functional study using a lineage-tracing mouse model, Tamoxifen was injected three times a week on postnatal weeks 6 and 7. Then, mice were injected with various conditions. First, 100 ng of TGF-β (240-B, R&D systems, USA) were injected intraperitoneal three times a week on postnatal week 8 or 12. TGF-β blocking antibody (10 mg/kg, BE0057, BioXCell, USA) was injected intraperitoneally three times a week from postnatal week. Scl-Ab (25 mg/kg, Amgen Inc., USA; Belgium) was injected twice a week on postnatal week 12 alone or with TGF-β or TGF-β blocking antibody to investigate the effects of combinatorial treatments. Mice were euthanized on postnatal weeks 8, 12, and 13 (baseline, prior to, and following treatment at the indicated time).

To investigate the regulatory effects of sclerostin, we used *SOST* transgenic mice (The Jackson Laboratory, USA) and femoral sections from *Sost* KO mice (kindly provided by Alexander Robling, Indiana University School of Medicine).

SOST-TG mice (The Jackson Laboratory, USA) were C57BL/6 mice introduced by human bacterial artificial chromosome (BAC; RM11-209M4) containing the human SOST gene, as well as DUSP3 and MEOX1 to regulate SOST expression by endogenous human regulatory elements. Mice were euthanized on postnatal week 8.

Except for spatially-resolved osteoblast-traced transcriptomics, femurs and tibiae were processed as follows. After harvest, all soft tissues were removed, and samples were rinsed twice with phosphate-buffered saline (PBS). Bones were fixed in 4% PFA overnight at 4 °C, washed three times with PBS, and decalcified with 10% EDTA for 2 weeks with gentle agitation. Following decalcification, samples were embedded in paraffin and sectioned for histological and immunohistochemical analyses.

All animal procedures were approved by the Institutional Animal Care and Use Committee of Seoul National University (approval no. SNU-220812-1).

### Spatially-resolved osteoblast-traced transcriptomics

*Dmp1*-CreERt2:mTmG mice were injected with tamoxifen five times starting at postnatal week 1 and euthanized at weeks 3, 5, and 6. Calvariae were dissected, embedded in OCT compound, sectioned at 8 μm thickness using a cryostat (CM1850, Leica, Germany), and mounted on sacrificial-layer-coated slides for SLACS.

SLACS, an in-house instrument used for target cell isolation, comprises optical and mechanical modules for the high-throughput retrieval of samples from the tissue.^11^ Fluorescent images were acquired (Ti2-E, Nikon, Japan), and regions expressing GFP were isolated using a near-infrared (*λ* = 1 064 nm) pulse laser, which vaporized the sacrificial layer beneath the target cells to isolate them into PCR tubes for subsequent reactions.

cDNA libraries were generated according to a previously published protocol.^11^ Isolated regions were incubated in lysis buffer at 50 °C for 1 h with 600 r/min shaking. Libraries were processed using Tn5 transposase and barcoded with Illumina adapters. Paired-end 150-bp sequencing was performed on an Illumina NextSeq platform, generating ~600 Mb per sample.

### Analysis of spatially-resolved osteoblast-traced transcriptomics

The sequencing reads were aligned against the mouse genome (GRCm39) using the STAR aligner with the default settings. The number of uniquely mapped reads of each sample was calculated using FeatureCounts with the default parameters for downstream analysis. The results from FeatureCounts were quantified and normalized using DESeq2,^42^ followed by differentially gene expression analysis. Only gene with a log_2_-fold change >1 and an adjusted *P*-value of <0.05 were considered positive differentially expressed genes. Gene Set Enrichment Analysis was performed using the R package fgsea^43^ on the normalized expression counts using DESeq2. Gene expression levels were mapped to corresponding pathways by the Molecular Signatures Database (MSigDB).^44^ The cell type proportions in each sample were estimated using CIBERSORTx with the default parameters.^45^ We used the result matrix obtained from FeatureCounts as input and the published scRNA-seq data as a signature matrix.

### Single-cell RNA-seq

*Dmp1*-CreERt2:mTmG mice (*n* = 4 per group) were sacrificed, and tibiae and femora were collected. The soft tissue was removed by scraping, and the epiphysis was cut off. Bone marrow cells were removed by centrifuging at 4 °C, 1 300 rcf for 5 min. The bones were crushed in ice-cold PBS Dulbecco’s solution (BY0511, BYLABS, South Korea) using scissors, a mortar and pestle and a razor blade. The crushed bone chips were washed four times with ice-cold DPBS and incubated with 5 mL of RBC lysis buffer (00-4333-57, eBioscience, USA) for 5 min. Then, the crushed bone chips were subjected to three serial digestions with 5 mL of digestion medium (2 mg/mL of collagenase type I (C0130, Sigma-Aldrich, USA) and 2 mg/mL of Dispase II (17105-041, Gibco, USA) in Hank’s balanced salt solution (14170112, Gibco, USA) at 37 °C 300 rcf for 20 min each. The collected supernatant was filtered through a 40 μm filter, and the digestion reaction was stopped by adding 15 mL chilled DPBS. After centrifugation of the stopped solution at 1 000 rcf for 5 min, the cell pellets were resuspended in 2% FACS buffer.

Before sorting, the cells were blocked with anti-mouse CD16/CD32 antibodies (TruStain FcX, 101319, Biolegend, USA) diluted 1:200 for 30 min on ice and stained with anti-mouse CD45 antibody (BD Horizon BV 421 Rat Anti-Mouse CD45 Clone 30-F11 (RUO), 563890, BD Biosciences, USA) diluted 1:100 for 1 h on ice to protect the tubes from light. CD45-negative cells were sorted (FACS AriaII, BD Biosciences, USA) into a new 1.5 mL tube containing 2% FACS buffer. Single cells were loaded onto a Chromium Controller (10X Genomics, USA). Libraries were constructed using Chromium Next GEM Single Cell 3’ RNA library v3.1 Reagent Kit according to the manufacturer’s protocol and sequenced.

### Analysis of scRNA-seq

Raw reads obtained from scRNA-seq were demultiplexed, aligned to the mouse genome (GRCm39) with the EGFP gene inserted, and collapsed into unique molecular identifiers (UMIs) with the CellRanger toolkit (version 8.0.0, 10x Genomics, USA). After generating count matrices from each experimental group using CellRanger, these matrices were integrated using SelectIntegrationFeatures, FindIntegrationAnchors, and IntegrateData in the Seurat package. The integrated matrix was processed using the Seurat pipeline.

### In vitro study using DBP

Demineralized bone papers (DBPs) were generated as previously described.^27,28^ Primary osteoprogenitors isolated from DsRed reporter mice (provided by B. Osborne, UMass Amherst) were seeded on DBPs and cultured for two weeks in osteogenic medium (α-MEM with 10% FBS, 1% P/S, 10 mmol/L β-glycerophosphate and 200 μmol/L L-ascorbic acid). Osteoblasts were then activated in normal medium (α-MEM with 10% FBS, 1% P/S) containing 10 nmol/L VD3, and 1 μmol/L PGE2. On day 20, VD3/PGE2 was withdrawn, and cells were cultured for an additional 6 days in normal medium with or without TGF-β (10 ng/mL). The conditioned media were collected on days 20, 23, and 26 and analyzed for OPG and RANKL using ELISA kits. On day 26, cells were fixed with 4% paraformaldehyde (PFA) for immunofluorescence analysis. For cytoskeletal visualization, F-actin filaments were stained with Alexa Fluor 488-conjugated phalloidin and nuclei were counterstained with DAPI. Images were acquired using a fluorescence microscope (EVOS), and cell alignment angles and vertical heights were measured using ImageJ.^28^ To access changes in osteoblast proliferation in response to TGF-β treatment, fixed cells were permeabilized with 0.1% Triton X-100 and blocked in PBS containing 10% goat serum and 1% bovine serum albumin (BSA) for 2 h. Cells were then incubated overnight at 4 °C with a primary rabbit anti-mouse Ki67 antibody (1:200) diluted in blocking solution. After three PBS washes, the cells were incubated with an Alexa Fluor 647-conjugated goat anti-rabbit secondary antibody (1:200) for 1 h. After additional PBS washes, images were obtained by fluorescence and confocal microscopy (A1R25, Nikon, Japan).^27^

For co-culture experiments, osteoblasts were first established on DBPs as described above to promote remineralization. After two weeks of mineralization, bone marrow monocytes (BMMs) isolated from eGFP reporter mice were seeded on top of osteoblast-remineralized DBPs and cultured in stimulation medium supplemented with 10 nmol/L VD3 and 1 µmol/L PGE2 to induce osteoclast differentiation for nine days. On day 23, VD3/PGE2 stimulation was withdrawn, and the medium was replaced with standard α-MEM containing or lacking TGF-β (10 ng/mL) for an additional six days.

For fluorescence imaging, osteoblasts (DsRed) and osteoclasts (eGFP) were monitored using a fluorescence microscope (EVOS) on days 0, 3, and 6 following the medium switch. On day 29, co-cultures were fixed with 4% paraformaldehyde for end-point analyses. TRAP staining was performed using a commercial kit according to the manufacturer’s instructions. To confirm multinucleated osteoclasts, cells were counterstained with DAPI for nuclear visualization. Osteoclast size was quantified as the total cytoplasmic area of multinucleated DAPI-stained cells using ImageJ software. To distinguish osteoclasts from undifferentiated precursors, only cells within a size range of 70–70 000 µm² were classified as osteoclasts.

### ELISA

Blood was collected via cardiac puncture before the animals were euthanized. Aliquots of serum were stored at −20 °C until use. Serum levels of P1NP, CTx1, TRAcP, OPG, RANKL, and TGF-β1 were measured via an enzyme-linked immunosorbent assay (ELISA) performed using ELISA kit for mouse P1NP (AC-33F1, Immunodiagnostic Systems, UK), mouse CTx1 (AC-06F1, Immunodiagnostic Systems, UK), mouse TRAcP 5b (SB-TR103, Immunodiagnostic Systems, UK), mouse OPG (MOP00, R&D systems, USA), mouse RANKL (MTR00, R&D systems, USA), and mouse TGF-β1 (DY1679-05 and DY007B, R&D systems, USA) according to the manufacturer’s instruction. Batch effects were corrected using the R Package ComBat (ver.3.10).^46^

Conditioned media samples were taken at different time points during the culture period. Levels of OPG and RANKL in conditioned media were measured via ELISA performed using an ELISA kit for mouse OPG (DY459, R&D systems, USA) and mouse RANKL (DY462, R&D systems, USA) according to the manufacturer’s instructions. Conditioned media samples were diluted to 1:30 in reagent diluent to ensure that the concentrations of OPG fell within the detection range of the assay.

### Hindlimb unloading experiments

Hindlimb unloading was performed according to a previously published protocol.^31^ In brief, tails of C57B1/6 J mice were pierced with a ring and suspended to reduce hindlimb weight-bearing. Two mice were housed per cage. From postnatal week 8, mice received treatments for 4 weeks. First, TGF-β blocking antibody (10 mg/kg, BE0057, BioXCell, USA) was intraperitoneally injected three times a week. Scl-Ab (25 mg/kg, Amgen Inc., USA; Belgium) was injected twice a week alone or with TGF-β blocking antibody to investigate the effects of combinatorial treatments. At week 12, the mice were euthanized. Serum was used for ELISA-based assessment of bone turnover markers. Micro-computed tomography analysis was performed on the femur, and femoral sections were analyzed by bone histomorphometry, TRAP staining, and immunohistochemistry.

### Immunohistochemistry staining

Formalin-fixed paraffin-embedded decalcified femur sections from 8-week-old mice were obtained. For anti-p-Smad3 immunohistochemistry staining, antigen retrieval was performed using citrate buffer (pH = 6.0, K8005, Dako, Denmark) at 95–97 °C for 30 min. Endogenous peroxidases were quenched, and slides were blocked in TNB buffer (Akoya Biosciences, USA), then stained with anti-phosphoSmad3 antibody (ab-51177, Abcam, UK) at a concentration of 1:200 overnight at 4 °C. Slides were washed and stained with the biotinylated secondary antibody (65-6140, Invitrogen, USA) at 1:2 000 for 1 h at room temperature. Slides were washed, incubated with horseradish peroxidase (HRP) coupled streptavidin (TS-000300, AKOYA Biosciences, USA) at 1:200 for 30 min, signals amplified using tyramide at 1:100 for 5 min, and HRP detection was performed using 3,3’-diaminobenzidine (DAB, SK-4100, Vector Laboratories, USA). Slides were briefly counterstained with hematoxylin before mounting. Quantification of p-Smad3+ osteocytes and osteoblasts was performed manually.

### Micro-computed tomography

Whole femurs of mice at postnatal week 12 across all experimental groups were harvested after euthanasia, fixed in a 4% PFA solution for 24 h, placed in 70% ethanol, and stored at 4 °C until imaging. The distal femur from each mouse was scanned using high-resolution μCT (SkyScan 1272; Bruker microCT, Kontich, Belgium) at 70 kV and 142 μA, with an isotropic voxel size of 5 μm using a 0.5-mm aluminum filter. Measurements were performed starting at 0.5 mm from the end of the primary spongiosa, including an area of 2 mm. The region of interest was obtained automatically using the “custom processing” function in CTAn (Bruker, Kontich, Belgium), which is a manufacturer-provided software. The three-dimensional images were reconstructed and displayed using the manufacturer-provided software CTvol (v.2.3.2.0). For both cortical and trabecular bone regions, the bone volume/total bone volume (BV/TV), thickness (Th), separation (Sp), and number (N) were assessed.

### Bone histomorphometric analysis

Eight and three days before euthanasia, the mice were intraperitoneally injected with calcein (10 mg/kg, #C0875, Sigma-Aldrich, Germany) and alizarin (20 mg/kg, #A3882, Sigma-Aldrich, Germany), respectively. Undecalcified femurs were fixed with formalin, dehydrated, and then embedded in methyl methacrylate (Sigma-Aldrich, USA), followed by sagittal sectioning. Calcein and alizarin labeling were fluorescence microscopy (Leica, Germany), and histomorphometric analysis of the bone compartment was conducted via Bioquant Osteo 2019 V19.9.60 program (Bioquant Osteo, USA).^47^ For Toluidine Blue staining, deparaffinized and rehydrated 3-μm sections were incubated in 0.04% Toluidine Blue (#198161, Sigma-Aldrich, Germany) solution for 10 min, followed by gentle rinsing in deionized water. Thereafter, the sections were dehydrated with ethanol and mounted with Permount™ Mounting Medium. Osteoblast number per bone surface (Ob.N/BS) Analysis followed the ASBMR guidelines. Both analysis was defined as the trabecular compartment located 1-2 mm distal to the growth plate.

In addition, deparaffinized tibia sections were used for tartrate-resistant acid phosphatase (TRAP) staining following the public protocols from the Center for Musculoskeletal Research at Massachusetts General Hospital.

### Statistical analyses

Gene expression data from both spatially resolved osteoblast-traced transcriptomics and scRNA-seq were analyzed using the R software (version 3.4.3). All measurements were obtained from at least three independent replicates and are presented as means ± standard error (SE) or as median ± 0.5 × interquartile range (IQR) with individual data points. Statistical significance was assessed using *t*-tests or Wilcoxon rank-sum tests, as indicated in the figures and legends. The representative images shown in the figures were validated by reproducing the results from at least three independent biological samples. No data points were excluded from the analysis. The animals were randomly assigned to the experimental groups to minimize selection bias.

## Supplementary information

Supplementary Figures

All data set related to figures

## Data Availability

All sequencing data were uploaded to the National Center for Biotechnology Information (www.ncbi.nlm.nih.gov/) under the accession number PRJNA1247789. All data associated with this study are presented in the paper or the Supplementary Materials. The source codes were available at https://github.com/ahyounchoi/Osteoblast.
