# Supplementary material for: Spatially resolved osteoblast-traced transcriptomics uncovers TGF-β as a combination target with sclerostin in osteoporosis
Source: Bone Res. 2026 Apr 2;14:37. doi: 10.1038/s41413-026-00521-9 (PMC13046724; doi:10.1038/s41413-026-00521-9)
Supplement: Supplementary file 1 — Supplementary Figures [file 41413_2026_521_MOESM1_ESM.pdf]

Spatially Resolved Osteoblast-Traced Transcriptomics Uncovers TGF-β as a Combination Target with Sclerostin in Osteoporosis

Ahyoun Choi *et al.*

*Correspondence: Sunghoon Kwon (skwon@snu.ac.kr)

and Sang Wan Kim (swkimmd@snu.ac.kr)

This supplementary material file includes:

Figs. S1 to S11

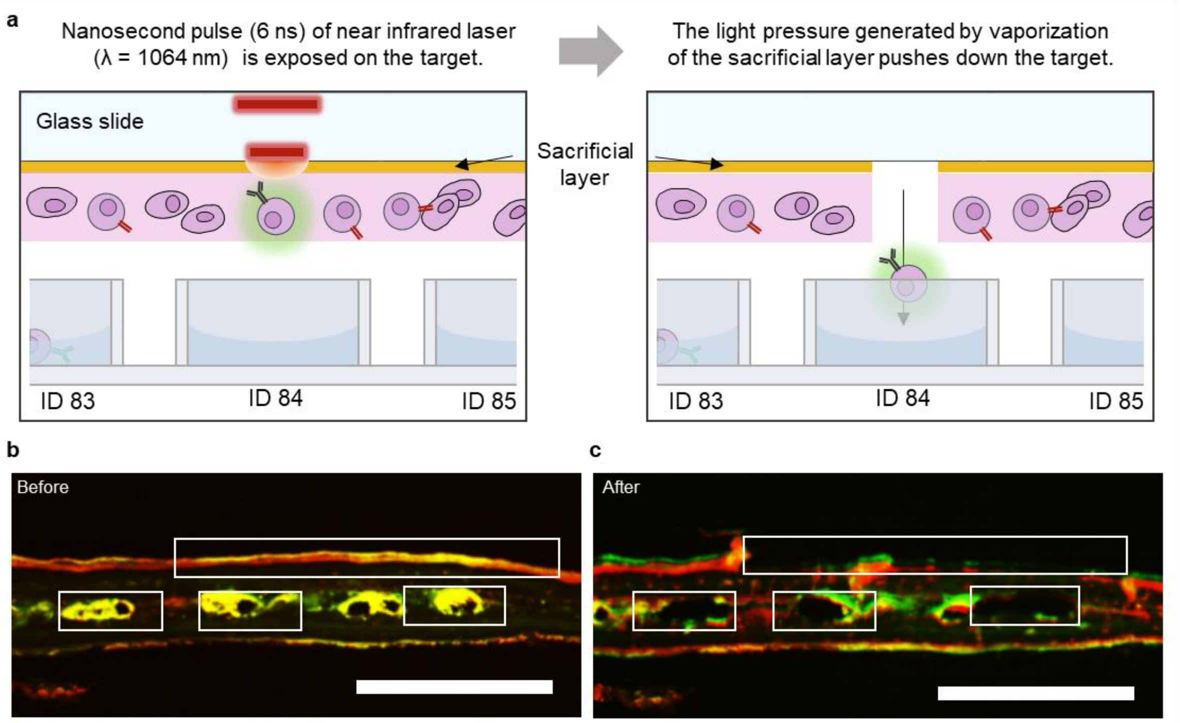

Fig. S1. SLACS enables precise enrichment of GFP+ cells on bone surface. a Schematic of SLACS-based isolation. Infrared laser irradiation vaporizes the sacrificial layer, enabling direct isolation of the target region. **b-c** Representative fluorescent images showing GFP and tdTomato expression in calvarial periosteum before (b) and after (c) target region isolation using SLACS. Scale bar = 50 μm.

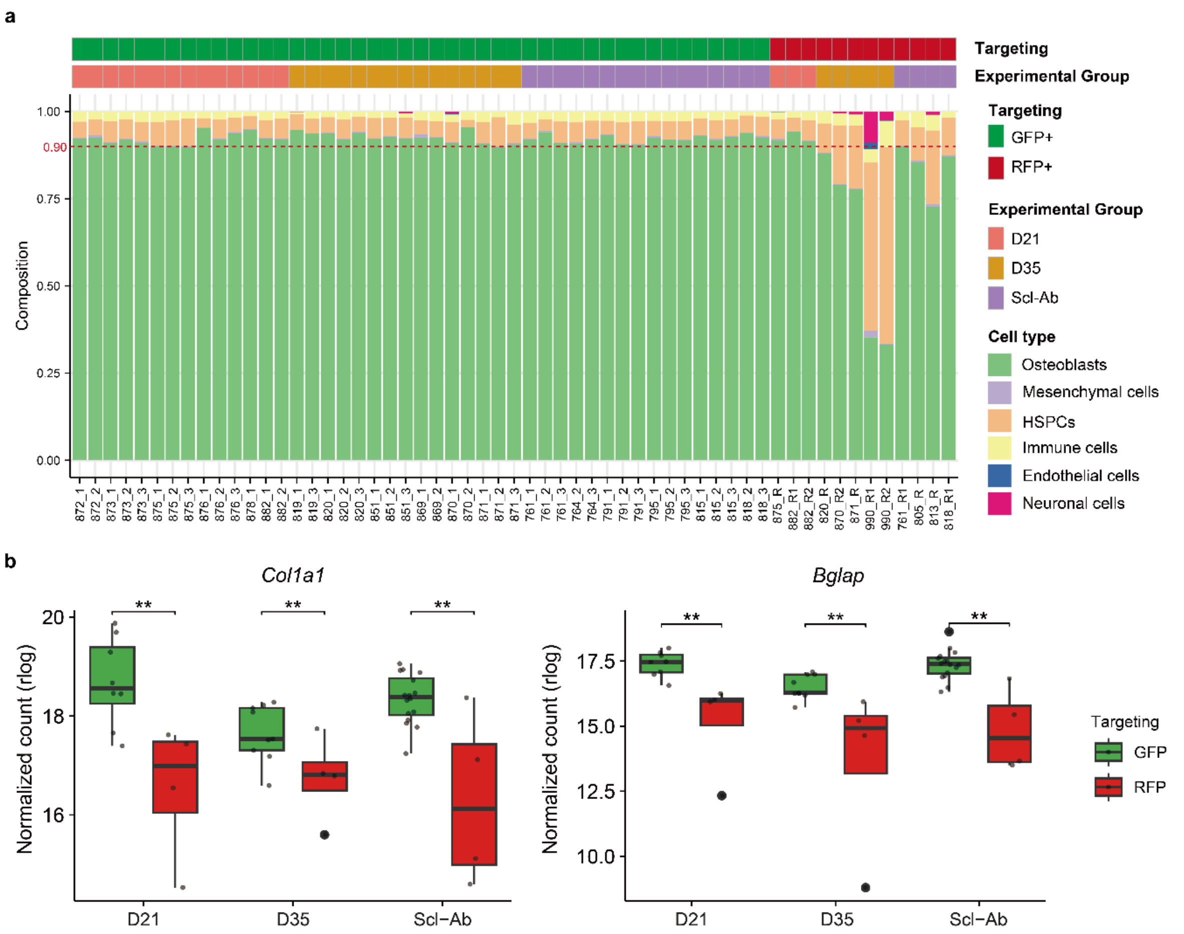

Fig. S2. The regions selected based on GFP are mostly composed of osteoblasts. a Stacked plot showing estimated cell-type proportions of each target region using CIBERSORTx with publicly available scRNA-seq data. Upper annotation represents targeting criteria; GFP+ or RFP+ regions. Bottom annotation represents experimental groups; D21, D35, and Scl-Ab treated. **b** Box plots showing regularized log (rlog) transformed expression of *Col1a1* and *Bglap*. Each point represents single region; Wilcoxon rank-sum test was used for statistics; ** $p < 0.01$

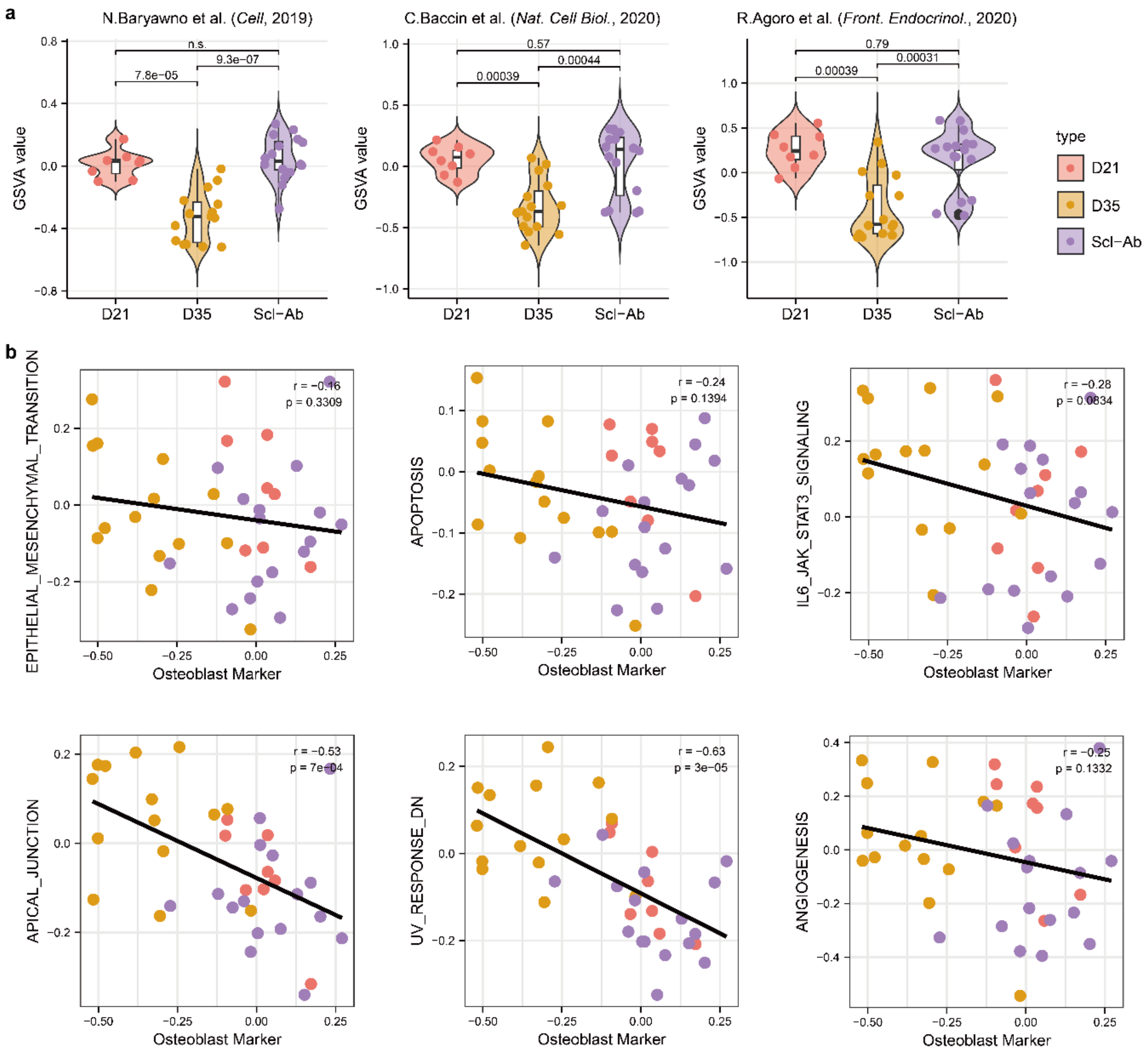

Fig. S3. Significant regulated pathways showed correlation with osteoblast marker scores. **a** Violin plots of GSEA scores using osteoblast DE gene sets from N. Baryawno et al. (2019), C. Baccin et al. (2020), and R. Agoro et al. (2020). Each point represents single region; Wilcoxon rank-sum test was used for statistics; n.s., not significant. **b** Scatter plot showing a correlation between significantly regulated pathways and osteoblast marker score. Pearson correlation coefficient (r) and p-value are calculated across regions.

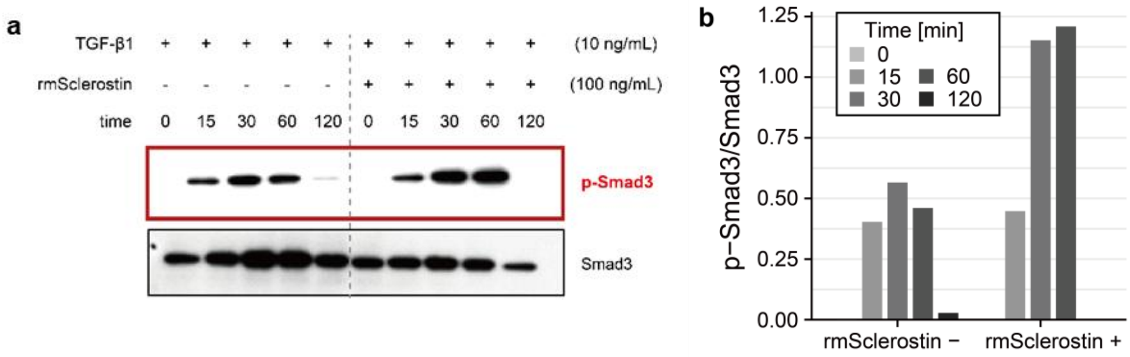

Fig. S4. TGF-β signaling is regulated by sclerostin modulation in the MC3T3-E1 cell line. **a** Western blot analysis of MC3T3-E1 cell extracts showing amounts of phosphorylated Smad3 (p-Smad3) and Smad3. MC3T3-E1 cell line culture in cell culture media containing TGF-β1 peptide (10 ng/mL) for induction of TGF-β signaling. Differences in the amount of p-Smad3 depending on treatment with or without recombinant mouse sclerostin (rmSclerostin, 100 ng/mL). **b** Bar plot showing TGF-β signaling activity shown as the ratio of p-Smad3 to Smad3, measured by ImageJ.

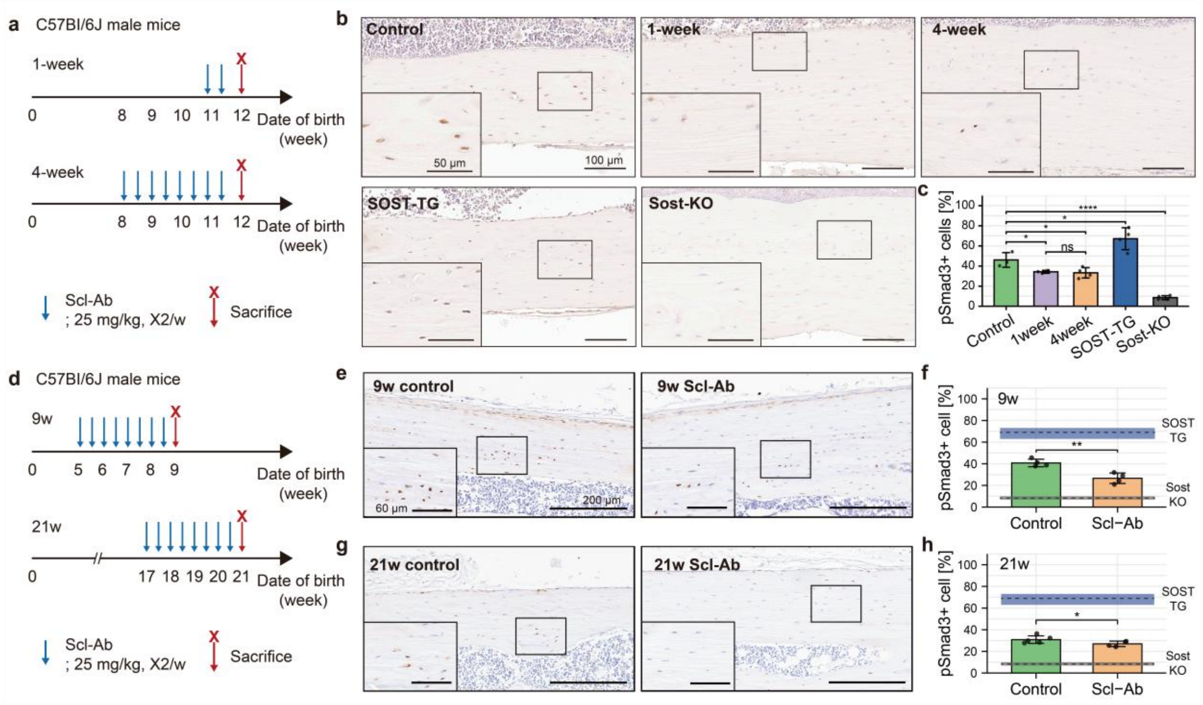

Fig. S5. Sclerostin inhibition downregulates TGF-β signaling at the protein level. a Experimental timeline of Scl-Ab treatment in C57BI/6J male mice. Mice received either short-term (1-week) or long-term (4-week) Scl-Ab administration and were euthanized 3 days after the final injection. **b** Representative images of p-Smad3 immunohistochemistry (IHC) in femoral sections. Scale bar = 100 μm and 50 μm. **c** Bar plot of quantification of p-Smad3+ cells relative to total counterstained cells. **d** Experimental timeline for age-dependent Scl-Ab treatment in C57BL/6J male mice. Mice at either 5 weeks or 17 weeks of age received 4 weeks of Scl-Ab administration and were euthanized 3 days after the final injection. **e** Representative images of p-Smad3 IHC in tibial sections from the 9w group. Scale bar = 200 μm and 60 μm. **f** Bar plot of quantification of p-Smad3+ cells relative to total counterstained cells for the 9w group. **g** Representative images of p-Smad3 IHC in tibial sections from the 21w group. Scale bar = 200 μm and 60 μm. **h** Bar plot of quantification of p-Smad3+ cells relative to total counterstained cells for the 21w group. All quantifications were performed using 4 to 6 mice per group. Data = mean ± standard deviation. Each point represents individual mouse. Statistical significance was assessed using the t-test; *, $p < 0.05$; **, $p < 0.01$; ns, not significant.

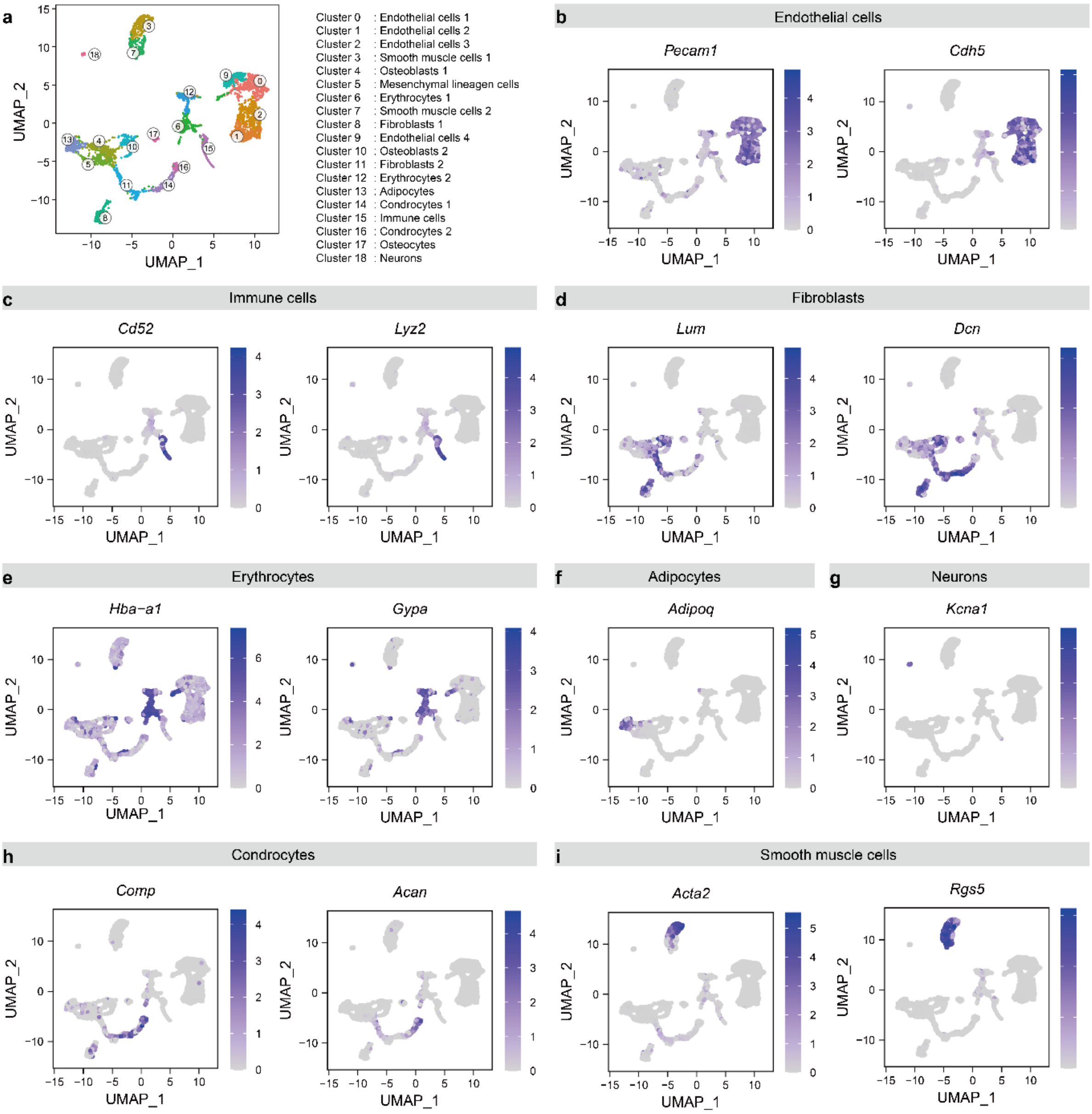

Fig. S6. Cell clusters are annotated based on known marker genes. a UMAP showing 19 clusters annotated using differential gene expression profiles and established marker genes. **b-i** Feature plots of representative marker genes used to identify major cell types: endothelial cells (*Pecam1* and *Cdh5*) (b); immune cells (*Cd52* and *Lyz2*) (c); fibroblasts (*Lum* and *Dcn*) (d); erythrocytes (*Hba-a1* and *Gypa*) (e); adipocytes (*Adipoq*) (f); neurons (*Kona1*) (g); chondrocyte (*Comp* and *Acan*) (h); and smooth muscle cells (*Acta2* and *Rgs5*) (i)

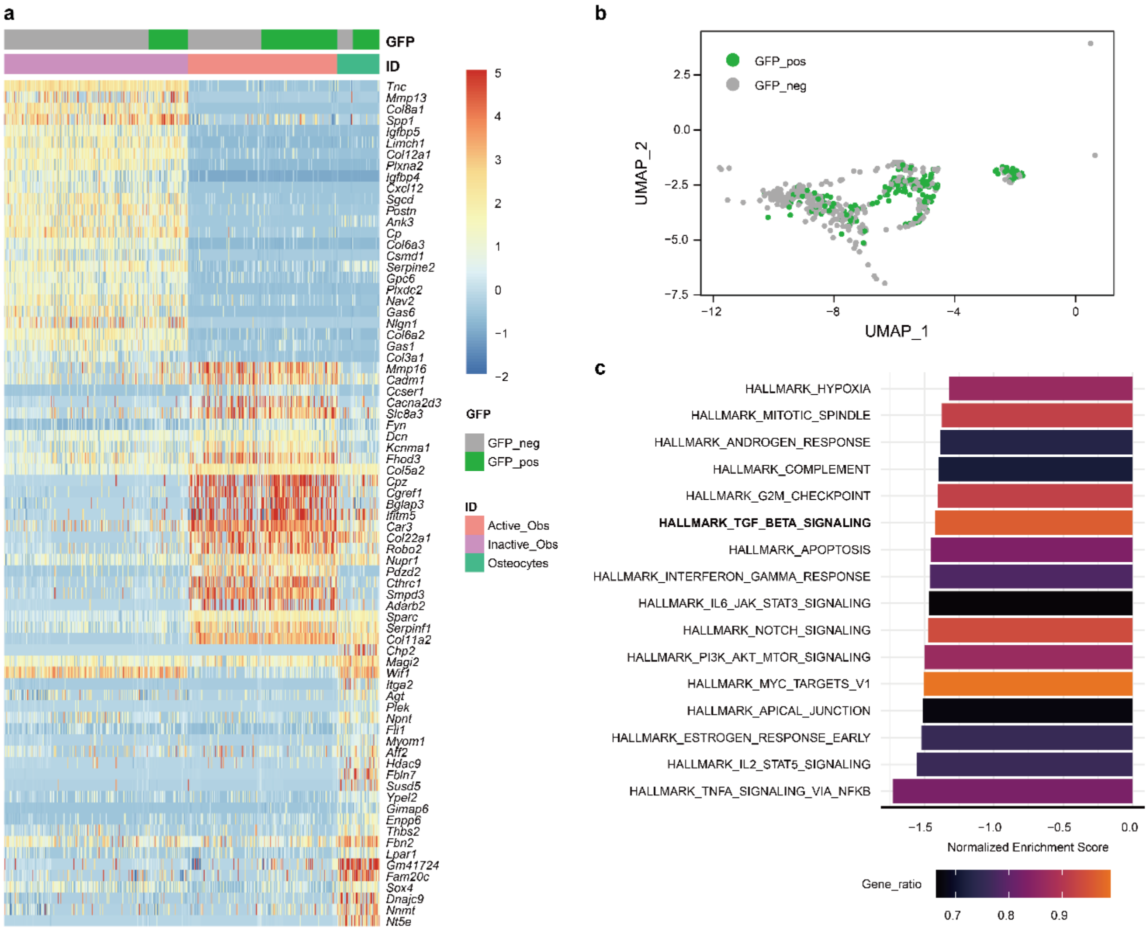

Fig. S7. scRNA-seq of cortical bone reveals distinct osteoblast-lineage cell clusters. a Heatmap showing the top 25 of significantly differentially expressed genes across individual osteoblast-lineage clusters; active osteoblasts, inactive osteoblasts, and osteocytes. **b** UMAP visualization of osteoblast-lineage clusters. Each data point represents an individual cell, and color indicates GFP expression cells: Green for the GFP expressing cells, and dark grey for the non-expressing cells. **c** Bar plots showing normalized enrichment scores (NES) of significant pathways identified by GSEA in active osteoblasts. Bar color indicates the gene ratio, representing the proportion of genes detected in the dataset relative to the total number of genes in each canonical gene set.

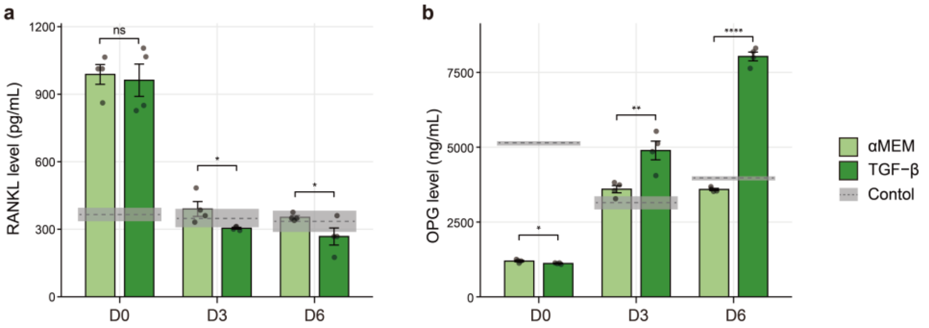

Fig. S8. Temporal changes in the expression of osteoblast state-related markers. a-b Bar plot of RANKL (a) and OPG (b) concentration across time points (n = 4). The grey bands (median ± 0.5×IQR) represent the control group without VD3 and PGE2 treatment. Each point represents individual well. t-test was used for statistics; *, $p < 0.05$; **, $p < 0.01$; ****, $p < 0.0001$; ns, not significant.

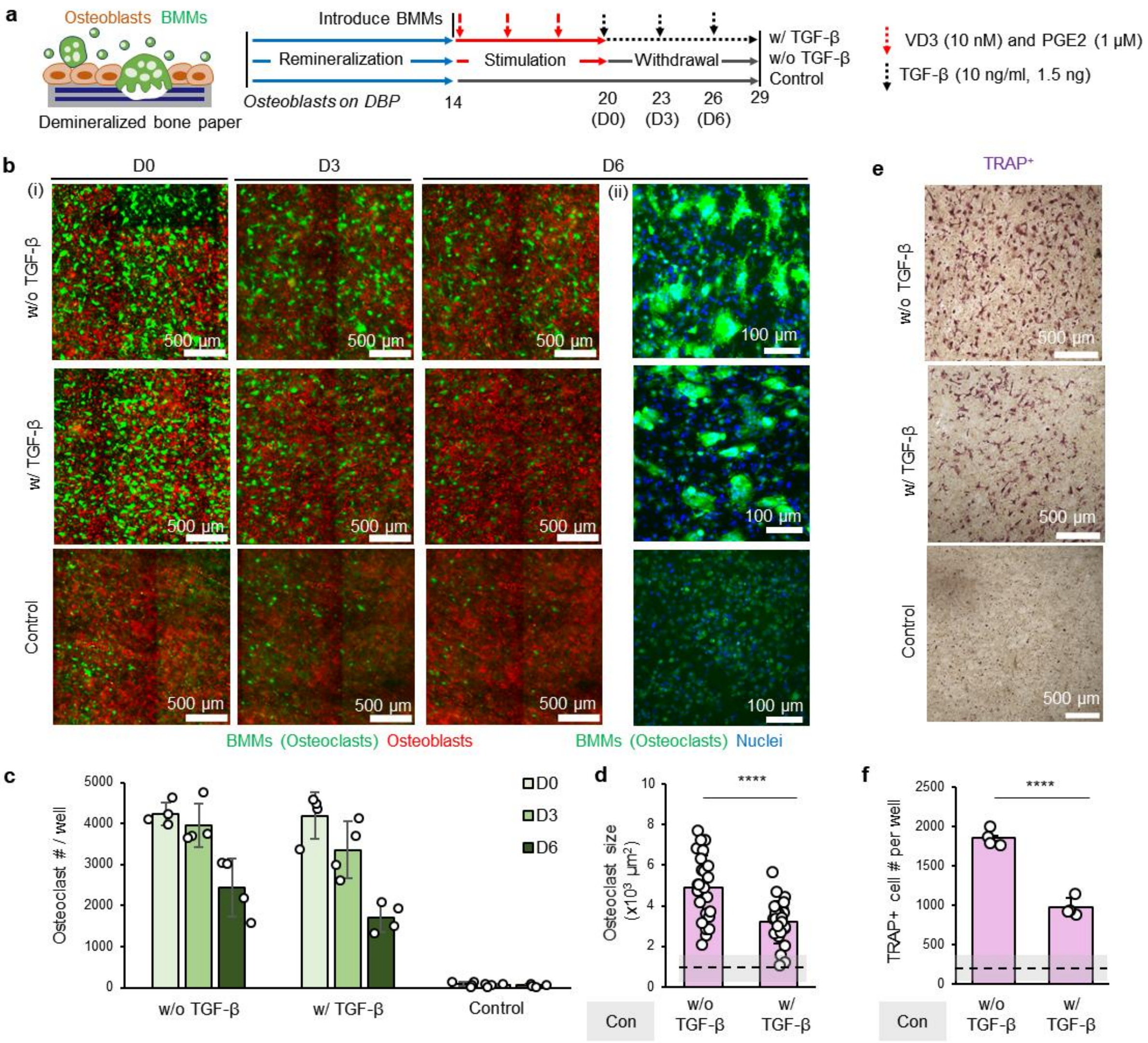

Fig. S9. Effect of TGF-β on active osteoblasts in a DBP-based bone organoid co-culture system. a Experimental timeline of osteoblast (DsRed)–bone marrow monocyte (BMM; eGFP) co-culture on DBP stimulated with VD3 and PGE2, followed by withdrawal with or without TGF-β to assess the effect of TGF-β on terminating osteoclast activity. **b** (i) Representative fluorescence images showing osteoblasts (red) and BMMs (green) at day 0 (D0), day 3 (D3), and day 6 (D6) after withdrawal of VD3/PGE2 stimulation under TGF-β, no TGF-β, or non-stimulated control conditions, illustrating a progressive decrease in large green multinucleated osteoclasts. (ii) Representative fluorescence images of multinucleated osteoclasts counterstained with DAPI (blue). **c** Time-course quantification of osteoclast density per well over six days (n = 4). **d** Quantification of osteoclast cell size at day 6 (n > 25). **e** Representative TRAP-stained (purple) images at day 6 under the indicated conditions. **f** Quantification of

TRAP$^+$ osteoclast counts per well (n = 4). Each point represents individual well. t-test was used for statistics; ****, $p < 0.0001$.

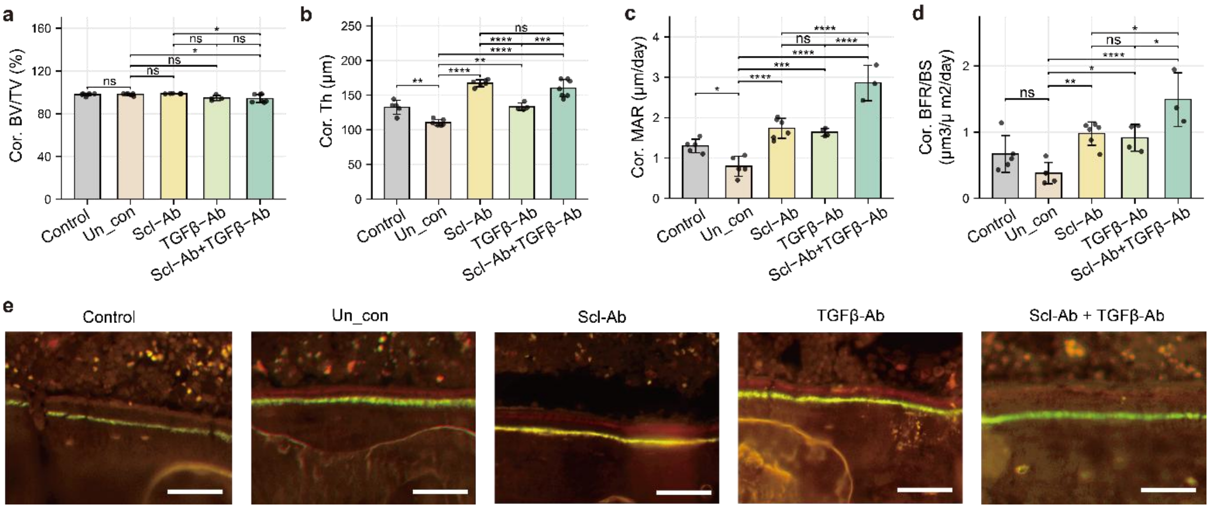

Fig. S10. Dual inhibition of TGF-β and sclerostin promotes endocortical bone formation in the hindlimb unloading model. Additional analyses related to endocortical regions are derived from the same experimental groups shown in Figure 6. **a-b** Bar plot of cortical bone volume/total volume (Cor. BV/TV, **a**), cortical thickness (Cor. Th, **b**) from µCT. Data = mean ± standard error. **c-d** Bar plot of endocortical bone formation rate per bone surface (Cor. BFR/BS, **c**) and mineral apposition rate (Cor. MAR, **d**) from labeling images. Data = mean ± standard error. Each point represents individual mouse. ANOVA was used for statistics; *, $p < 0.05$; **, $p < 0.01$; ***, $p < 0.001$; ****, $p < 0.0001$; ns, not significant. **e** Representative confocal images of endocortical surface in femoral bone sections showing calcein (green), alizarin (red) signals, and DAPI (blue). Scale bar = 100 µm.

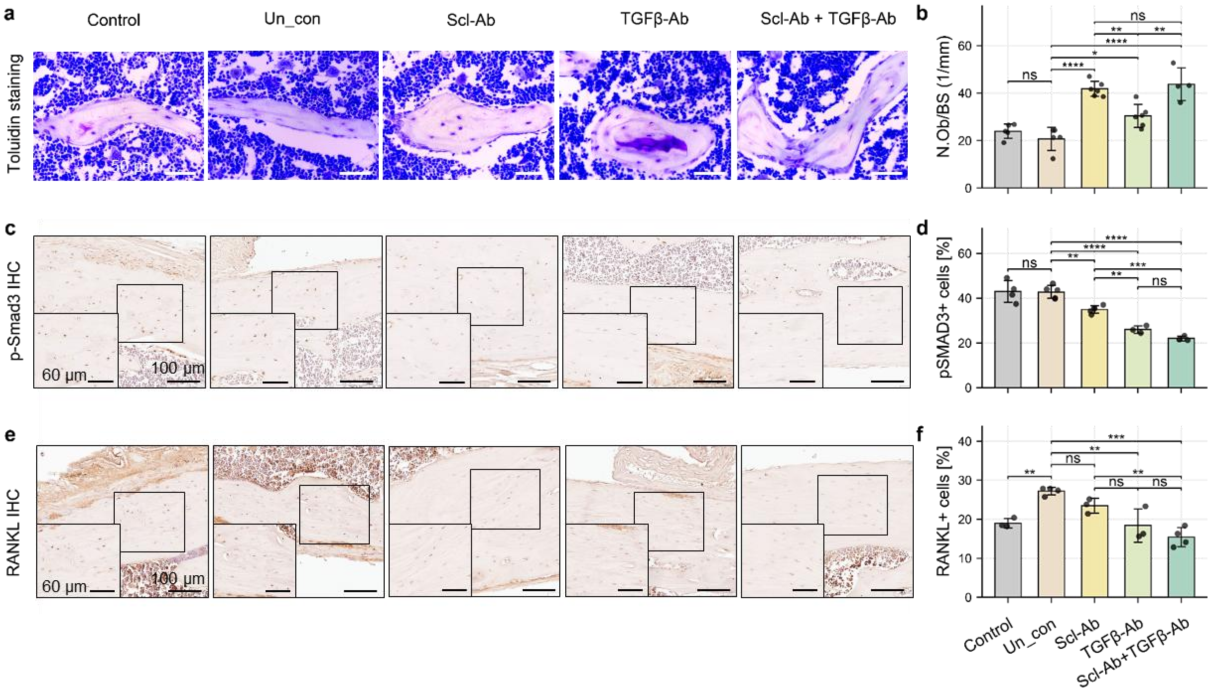

Fig. S11. Underlying mechanisms related to co-inhibition. Additional analyses are derived from the same experimental groups shown in Fig. 6. **a** Representative toluidine blue staining images in femoral sections. Scale bar = 50 μm. **b** Bar plot of quantification of osteoblasts per bone surface (BS). **c** Representative images of p-Smad3 IHC in tibial sections. **d** Bar plot of quantification of p-Smad3+ cells relative to total counterstained cells. **e** Representative images of RANKL IHC in tibial sections. **f** Bar plot of quantification of RANKL + cells relative to total counterstained cells. All quantifications were performed using 3 to 6 mice per group. Scale bar = 100 μm and 60 μm. Data = mean ± standard error. Each point represents individual mouse. ANOVA was used for statistics.; *, $p < 0.05$; **, $p < 0.01$; ns, not significant.
